# Use of Soluble Extracellular Regions of MmpL (SERoM) as Vaccines for Tuberculosis

**DOI:** 10.1038/s41598-018-23893-3

**Published:** 2018-04-04

**Authors:** Emily J. Strong, Nicholas P. West

**Affiliations:** 1 School of Chemistry and Molecular Biosciences, University of Queensland, Brisbane, 4067 Australia; 2 Australian Infectious Disease Research Centre, University of Queensland, Brisbane, 4067 Australia

## Abstract

The current vaccine for tuberculosis (TB) is a live attenuated strain of *Mycobacterium bovis* (BCG) and while effective at reducing the potential for disseminated TB in young children its disease protection rates in adults is highly variable while it confers little protection against latent TB. With these limitations a new vaccine is desperately needed. We investigated the efficacy of three members of the mycobacterial membrane protein Large (MmpL) family as potential subunit vaccines for TB. MmpLs are large, multifunctional integral membrane proteins, and as such are recalcitrant to purification. Here, we describe a strategy of producing synthetic antigens comprised of the soluble, extracellular regions of MmpL (SERoM)-1, MmpL8 and MmpL10 (SERoM-8 and 10 respectively) as potential vaccine candidates. SERoM-1 and SERoM-8 were determined to be highly immunogenic by IFN-γ ELISpot assays, with 0.1% of all splenocytes from SERoM-1 vaccinated mice producing IFN-γ when re-stimulated with MmpL1. A combined SERoM-1, −8 and −10 vaccine demonstrated significant protection against *M. tuberculosis* challenge in a murine model of TB, resulting in approximately 10-fold reduction in bacterial numbers following challenge in both the lungs and spleens compared to adjuvant only vaccinated mice. These protective effects were comparable to that achieved with BCG.

## Introduction

Tuberculosis (TB) currently accounts for more human death than any other infectious disease. With 10.4 million new cases of TB in 2015, resulting in 1.8 million deaths. One third of the world’s population is believed to be latently infected with *M. tuberculosis* and approximately 10% of these immunocompetent latent patients will reactivate with active TB, thus perpetuating the disease globally^1^.

The only vaccine ever licensed to protect against TB is the attenuated *M. bovis* Bacillus Calmette-Guérin (BCG) live vaccine. There are up to 16 regions of deletions in modern BCG strains, accounting for up to 140 genes, although not all strains contain all regions of deletions^2^. BCG is one of the most administered vaccines in history and is still being administered to 85% of infants despite its controversial efficacy reported to range from 0 to 80%^3^. It is currently accepted that BCG protects against disseminated TB in young children^1^. As TB is still a disease of significant global impact it is evident that the current vaccine remains limited in its potential to address adult TB and its transmission. A new vaccine is urgently needed.

Many of the current strategies for the development of new vaccines against *M. tuberculosis* rely on the use of known highly immunogenic and soluble proteins secreted by *M. tuberculosis* such as antigen 85 and ESAT-6^4^. The use of various adjuvant systems or vaccine delivery systems to improve the ability to target the Th1 immune pathway in order to create a standalone subunit vaccine represents an important aspect to TB vaccine development^5–10^. The dimethyl dioctadecyl ammonium bromide (DDA) and monophosphoryl lipid A (MPLA) adjuvant system is well established for its ability to trigger a pro-inflammatory cytokine response, recruiting macrophages and neutrophils and subsequently initiating a Th1 immune response^11^, making it an appropriate choice as adjuvant system for the present study.

The MmpL proteins possess extended surface domains which are involved in the translocation of cell wall outer components and sequestration of essential nutrients from the host. Their extracellular location and important functionality make them strong potential vaccine candidates. MmpL1, MmpL8 and MmpL10 are family members with known roles in *M. tuberculosis* pathogenesis. *MmpL1* is a functional, single open reading frame in *M. tuberculosis* but contains a stop codon insertion resulting in a truncated transcript in *M. bovis*, the parental stain of the BCG vaccine. This could indicate that full MmpL1 is not expressed in BCG and thus unavailable to the host for the development an anti-MmpL1 adaptive immune response^12^. Both MmpL8 and MmpL10 have been associated with host interactions during infection. *M. tuberculosis* strains containing mutations in *mmpL8* and *mmpL10* have severely attenuated growth in the lungs of mice during the early stages of *M. tuberculosis* infection. In a murine infection it was observed that MmpL8 may play a role in the suppression of Th1 immunity^13^. These findings indicate a strong requirement for their *in vivo* expression and forms the basis for our interest in investigating their potential as subunit vaccine candidates.

It is well documented that multivariant subunit vaccines can confer greater protection than single variant vaccines^8,14–16^. Clearly, as a live attenuated bacterial vaccine, BCG offers hundreds of protein antigens to induce protective immunity^17^. Whilst subunit vaccines are not intended to offer this breadth of antigens, careful selection of a few highly immunogenic antigens have proved eficacious^14,18–20^. T cell epitopes of *M. tuberculosis* are considered to be suitable, and necessary, potential inclusions as subunit vaccine candidates. In Addition to B-cell epitopes, MmpLs contain strong T-cell epitopes within the predicted host exposed region of the protein^21^.

This study examined the immunogenicity of synthetic di-polypeptides incorporating the soluble extracellular regions of MmpL (SERoM) proteins of individual MmpL transporter proteins. Further examination of the protection achieved by SERoM subunit vaccines derived from MmpL1, 8 and 10 when administered in a DDA/MPLA adjuvant system was also examined. The immunogenicity of SERoM was tested using ELISpot assay to determine the number of IFN-γ splenocytes. The protection conferred by these vaccines was tested by aerosol challenge with *M. tuberculosis* after vaccination and subsequent analysis of bacterial loads in the lungs and spleens of infected mice. This study reveals that SERoM1 and SERoM8 are immunogenic proteins, with SERoM1 providing protective efficacy against *M. tuberculosis* infection. Finally, a combined SERoM vaccine confers strong and enhanced protection in mice.

## Results

### SERoM Vaccine Construction and Purification

**Figure 1:**
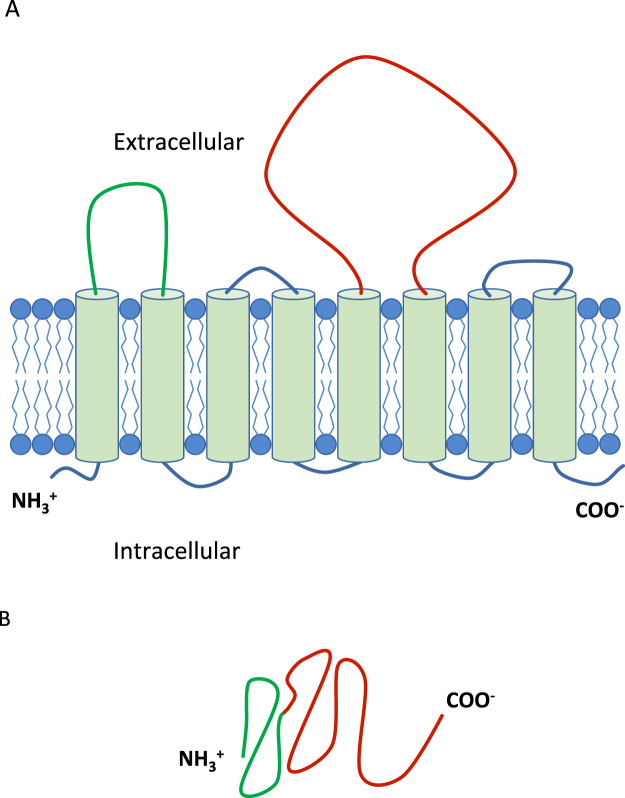
Illustration of SERoM di-polypeptide construction strategy. (**A**) Two dimensional depiction of an example MmpL protein. SERoM regions highlighted in green (R1) and red (R2). Transmembrane regions (8 of 12 shown) shown as barrels. (**B**) Example structure of SERoM di-polypeptide fusion vaccine, comprised of extracellular regions R1 and R2.

**Figure 2:**
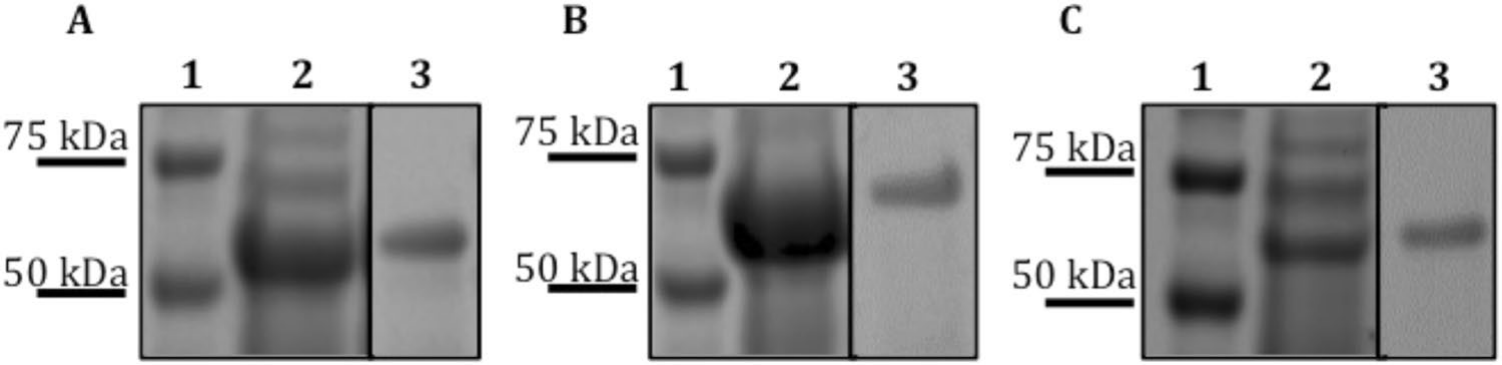
SDS-PAGE analysis of purified recombinant vaccine antigens. Lane 1, Molecular Wight Marker; Lane 2, Clarified whole cell lysate of *E. coli* expressing strain; Lane 3, IMAC purified, refolded soluble MmpL polypeptide. (**A**) SERoM1, expected size 58 kDa. (**B**) SERoM8, expected size 61 kDa (**C)** SERoM10, Expected size 69 kDa. Images A, B and C are cropped from originals (Supplementary Figures 2 and 3) for clarity and brevity.

The selection of multiple proteins increases the possibility of identifying an antigenic T-cell epitopes. We were interested in increasing the breadth of the antigenic repertoire while minimising the peptide size and maximising protein solubility. While the transmembrane domains of the MmpL proteins show a high level of similarity of 72–75% as determined by multiple sequence alignment^22,23^ (Table 1), the extracellular domains of MmpL1, MmpL8 and MmpL10, as identified by TMHMM^24,25^, were significantly more variable. The genome regions encoding the two major extracellular loops of each protein were cloned in-frame into an *E. coli* protein expression vector to create dimeric polypeptides (Fig. 1). The amino acids mapping to these regions for MmpL1 are inclusive of residue 40 to 192 (R1) and residue 396 to 759 (R2); for MmpL8, residue 65 to 219 (R1) and residue 423 to 872 (R2); and for MmpL10, residue 1 to 170 (R1) and residue 379 to 804 (R2). These engineered dimeric polypeptide proteins were expressed in and purified from *E. coli* (Fig. 2).

**Table 1:** Similarity comparison of MmpL1, MmpL8 and MmpL10 regions.

	Extracellular Regions	Transmembrane Regions	Whole Protein
MmpL1/MmpL8	25.70%	72.30%	28.14%
MmpL1/MmpL10	26.47%	72.31%	29.14%
MmpL8/MmpL10	52.05%	75.19%	58.04%

While the use of individual T-cell epitopes of known mycobacterial antigens has been previously tested in subunit vaccine formulations^18^, the use of soluble regions from otherwise wholly insoluble membrane proteins is a novel method for vaccine development against TB. Excluding the large hydrophobic regions of the proteins enabled the use of *E. coli* as an expression system for the production of large quantities of the desired soluble regions of the MmpL proteins. Furthermore, this selective expression strategy significantly reduced the size of these proteins, allowing for simplified expression conditions and handling as fusion proteins (Fig. 2).

### SERoM Immunogenicity

**Figure 3:**
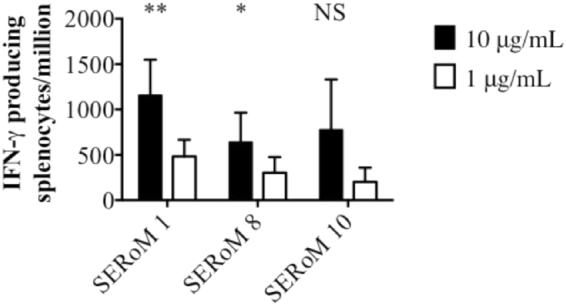
Immunogenicity of SERoM vaccines in C57BL/6 mice as measured by number of IFN-γ producing splenocytes. Mice (n = 3) were vaccinated subcutaneously three times with SERoM vaccines. Mean number of IFN-γ producing splenocytes per million cells, corrected for background (media only) is shown together with SD. Significance was calculated compared to relevant adjuvant vaccinated control via multiple unpaired, two tailed t-tests. *p < 0.05, **p < 0.01. Results representative of duplicate experiments.

To determine the immunogenicity of SERoM vaccines ELISpot assays were conducted to determine the number of IFN-γ producing splenocytes in spleens of vaccinated mice compared to mice vaccinated with adjuvant only (Fig. 3). The number of IFN-γ producing splenocytes from SERoM1 and SERoM8 vaccinated mice was significantly increased compared with adjuvant controls when stimulated with 10 µg/mL of vaccine antigen. Specifically, very robust responses were observed for SERoM vaccinated mice, which resulted in over 1000 cells per million, equating to 0.1% of all splenocytes, produced IFN-γ when stimulated with 10 µg/ml of purified SERoM1. The SERoM10 vaccinated mice produced a large, number of IFN-γ producing splenocytes although this result did not reach statistical significance. Production of IFN-γ from splenocytes showed a dose response when stimulated with 10 µg/mL and 1 µg/mL of recall antigen for all three vaccine groups (Fig. 3)

### Protective Effects of SERoM Vaccines

**Figure 4:**
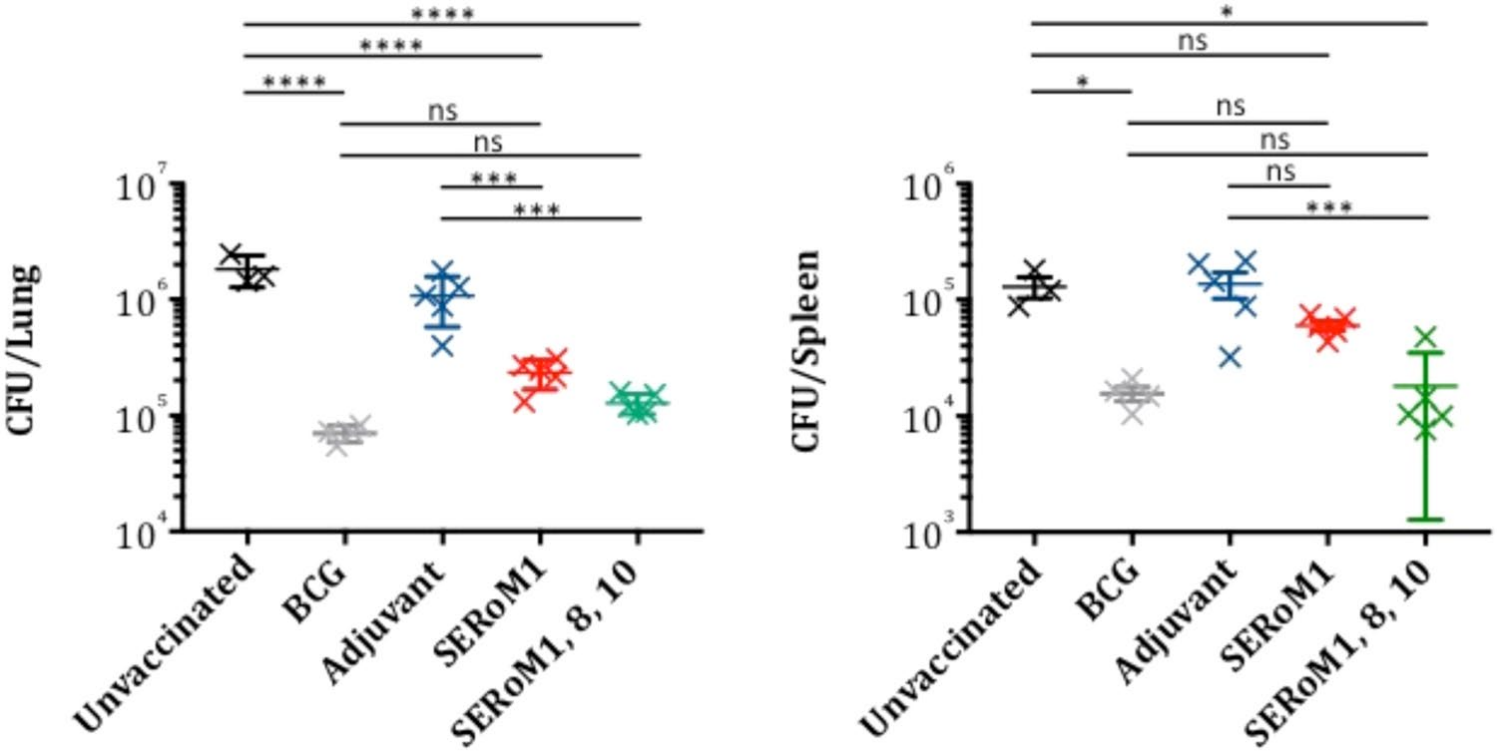
Protective effects of SERoM vaccines in mice. C57BL/6 Mice (n = 5) were vaccinated subcutaneously three times with SERoM vaccines, adjuvanted with DDA/MPLA. Mice were subsequently aerosol challenged with M. tuberculosis H37Rv 6 weeks post third vaccination. (**A**) protection in the lung of vaccinated mice. (**B**) protection in the spleen of vaccinated mice. Crosses are individual mice with mean and standard deviation shown. Significance was calculated compared to relevant control group via one-way ANOVA with Tukey’s multiple comparisons test. *p < 0.05, ***p < 0.005, ****p ≤ 0.0001.

As SERoM1 was the most immunogenic vaccine it was selected as the primary SERoM as a sole vaccine for analysis of protective efficacy against *M. tuberculosis* challenge. However, as single antigen TB vaccines are unlikely to be efficacious in all individuals, and many studies have shown that the inclusion of multiple antigens in a subunit vaccine can greatly improve its efficacy^8,16,19^, we also examined whether an enhanced protective effect could be achieve with a combined SERoM vaccine. For this arm of the study we combined equal quantities of SERoM1, 8 and 10 into a single formulation with DDA/MPL. Mice were subcutaneously vaccinated 3 times at fortnightly intervals with SERoM or adjuvant only and rested for 6 weeks prior to aerosol challenge with virulent *M. tuberculosis*. SERoM1 vaccinated mice showed a strong and significant protective effect in the lung, exhibiting a significant 0.8 log10 reduction in bacterial numbers compared to adjuvant only vaccinated mice (Fig. 4). A similar reduction in colonisation was also observed in the spleen, however statistical significance was not achieved.

**Figure 5:**
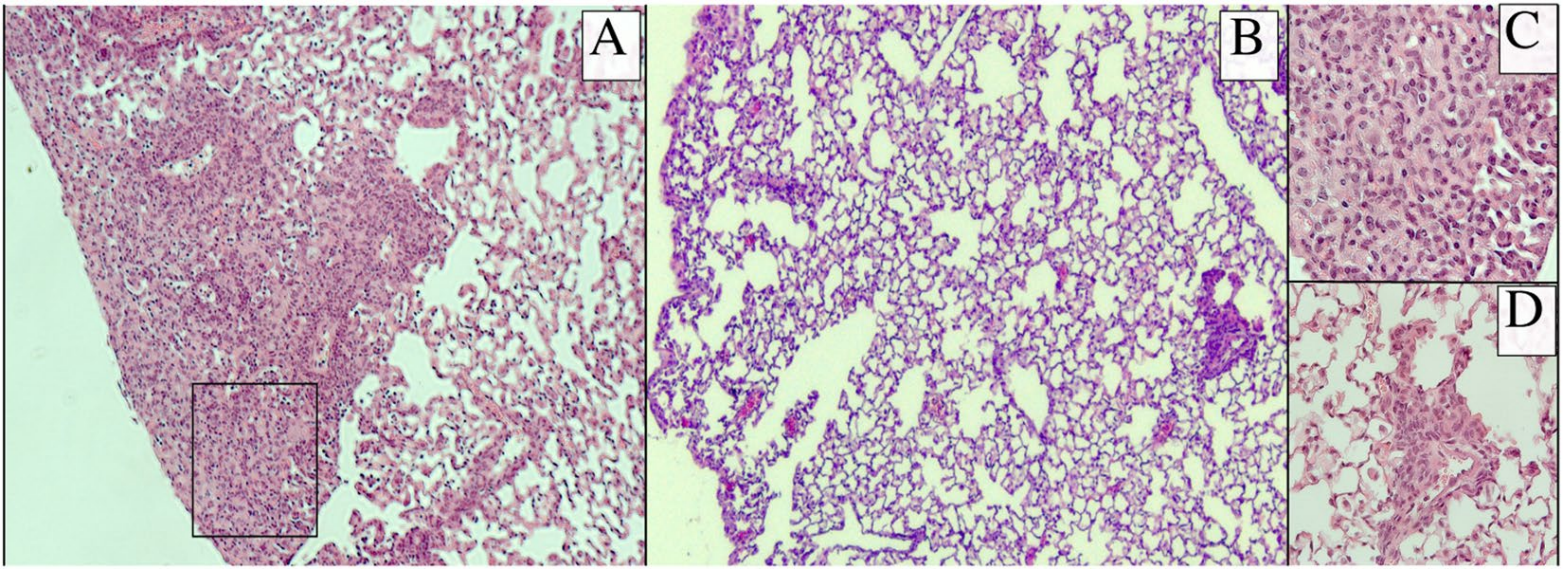
Lung pathology of *M. tuberculosis* infected mice. Lungs collected four weeks after *M. tuberculosis* challenge were fixed in formalin, paraffin embedded and subject to H&E staining. (**A**) low magnification micrograph of adjuvant only vaccinated mice. High magnification micrograph of boxed region shown in (**C**). (**B**) low magnification micrograph of SERoM 1, 8, 10 cocktail vaccinated mice. High magnification of small inflammatory lesion shown in (**D**).

A very strong, 1-log reduction in bacterial load was observed in the lungs of mice vaccinated with the combination SERoM vaccine compared to adjuvant only vaccinated controls. Additionally the combination SERoM vaccine achieved a strong protective effect in the spleens of vaccinated animals, also with a 1-log10 reduction. Indeed, this protective effect reached levels of protection not significantly different from that achieved by BCG (Fig. 4); an outcome rarely observed with subunit vaccines. The protective effects of SERoM vaccine cocktail is also evident from lung pathology examination. Whereas adjuvant only vaccinated mice were observed to have multiple, large lung lesions of polycellular infiltrate (macrophages, neutrophils and lymphocytes) (Fig. 5A and C), mice vaccinated with SERoM1, 8 and 10 displayed a largely normal lung pathology with only few small inflammatory lesions (Fig. 5B and D).

## Discussion

Subunit vaccines are well established in the literature as being immunostimulatory and even conferring a protective effect against *M. tuberculosis* infection^8^. While it is unlikely protein subunit vaccines will be utilised as single antigen formulations to replace the live, multivalent BCG as a priming vaccine^17^, subunit vaccines show great potential as boosting vaccines administered to evoke a targeted memory response in BCG immunised individuals, particularly when multivalent in nature. Furthermore, subunit vaccines have the added benefit of improved safety as opposed to live formulations. This study examined the potential of MmpL proteins as subunit vaccine candidates with a specific emphasis on the soluble surface exposed regions. The SERoM1 and SERoM8 synthetic vaccines were found to be significantly immunogenic with SERoM1 conferring reproducible protection against H37Rv infection in C57BL/6 mice compared to unvaccinated controls. A very significant level of protection against *M. tuberculosis* challenge was achieved following vaccination with the SERoM combination vaccine, in both the lungs and the spleen.

Fusion protein vaccines have been demonstrated to elicit enhanced protection compared to that of their component parts. A cutinase like protein (Culp) -fusion vaccine of Culp-1 and Culp6 in DDA/MPLA provided nearly 1 log protection in C57BL/6 vaccinated mice after an aerosol *M. tuberculosis* infection whereas Culp1 and Culp6 delivered as individual vaccines only conferred a 0.3 to 0.5 log protection respectively^19^. Similar results were observed with an ESAT-6/Ag85B fusion protein vaccine delivered in DDA/MPLA to C57BL/6 mice. Additionally the ESAT/Ag85B fusion stimulated long term immunity whereas in BCG vaccinated mice protection had waned^20^. Furthermore, the delivery of antigens as fusions, rather than antigen cocktails has been demonstrated to provide significantly enhanced protection to guinea pigs from an aerosol challenge of *M. tuberculosis*^26^. Development of SERoM antigens into single polypeptide fusion proteins may provide further efficacy and thus further validation of our approach.

The DDA/MPLA adjuvant formulation has been shown here, and in other studies, to be very effective at stimulating protective responses towards *M. tuberculosis* antigens. It is interesting to note however, that in both the lung and spleen of adjuvant-only vaccinated mice some non-specific protection was observed against *M. tuberculosis* infection at four weeks post vaccination (Fig. S1). Although this response did not reach statistical significance, this is an important observation and needs to be considered when using the DDA/MPLA adjuvant system for *M. tuberculosis* vaccine trials. As shown in Fig. 4, all non-specific protection had waned by 6 weeks post vaccination and we would therefore recommend these conditions for future testing with this system, as we have in the present study.

The inclusion of multiple immunogenic peptides clearly increases the efficacy, and efficiency of subunit vaccines, but the identification of the most appropriate antigens and their combinations may yet still need to be discovered. The exclusion of naturally insoluble proteins from such vaccine trials in the past may have hampered this effort, concealing epitopes of potential therapeutic value from testing. Integral membrane proteins are good examples of such antigens and our strategy of vaccinating with just the soluble regions of otherwise insoluble proteins opens up the discipline to the production and testing of a great many *M. tuberculosis* membrane proteins, raising the potential for many more protective T cell epitopes to be discovered. We have demonstrated this principle, as well as the protective capacity of antigens derived from important cell wall transporters of *M. tuberculosis*, i.e. MmpL1, MmpL8 and MmpL10, together with the enhanced protective effects of combination multivalent vaccines, but for the first time, as a novel formulation strategy.

## Methods

### Reagents

All reagents used were analytical grade unless otherwise stated and stored as specified on the technical data sheets and safety manuals. Unless otherwise stated all reagents and chemicals were sourced from Sigma-Aldrich.

### Bacterial Strain and Growth Conditions

*M. tuberculosis* (H37Rv) and BCG (pasteur)  strains were cultured on Middlebrook 7H11 agar (Difco) supplemented with 0.5% v/v glycerol and 10% v/v OADC (0.02% oleic acid w/v, 5% BSA w/v, 2% sucrose w/v, 0.002% catalase w/v and 0.85% NaCl w/v). All cultures were incubated at 37 °C for two to three weeks. Single cell suspensions were prepared by centrifuging at 800 × g for 8 min. *E. coli* BL21(DE3) was used for protein expression and cultured in Luria Bertani (LB) broth (Difco) and cultured on LB supplemented with 15 g/L of bacteriological agar. When required media were supplemented with ampicillin at 100 µg/mL. All cultures were incubated at 37 °C. *E. coli* broth cultures were incubated with orbital agitation at 200 rpm.

### Protein expression and purification

Synthetic MmpL peptide regions were designed using TMHMM^25^. The coding regions for the two large loops found in RND proteins as predicted by TMHMM were provided to Genescript (New Jersey, USA) for synthesis and codon optimisation. *E. coli* codon optimised sequence was prepared into vector pET19b for expression in *E. coli* BL21(DE3). Proteins were expressed by induction with 0.5 mM IPTG in LB broth. Inclusion bodies were separated from bacterial cells and protein purified using immobilised metal affinity chromatography. Inclusion bodies were separated by collecting *E. coli* BL21(DE3) at 4,000 × *g* for 20 min. Cell pellets were resuspended in 10 mL resuspension buffer (50 mM HEPES pH7.5, 0.5 M NaCl, 10 mM EDTA, 5 mM DTT, 0.35 mg/mL lysozyme and protease inhibitor (cOmplete protease inhibitor cocktail tablets, Roche) and incubated at room temperature for 30 min. A final concentration of 1% Triton-X-100 was added and cells lysed by freeze thawing. DNA was sheared using sonication and further incubated for 1 hour at room temperature with gentle agitation in 60 units DNAseI (NEB) and 5 mM MgCl_2_. Inclusion bodies were pelleted by centrifugation in a Beckman Avanti J centrifuge at 30,000 × *g* for 30 min at 4 °C and subsequently washed in PBS containing 1% Triton-X-100 and 1 mM EDTA. Inclusion bodies were again pelleted and subsequently washed in PBS containing 1% Triton-X-100. Inclusion bodies were pelleted again and resuspended overnight in two mL of solubilisation buffer (50 mM HEPES pH 7.5, 0.5 M NaCl and 6 M guanidine-HCl) at 4 °C.

Recombinant protein was purified using TALON (Clonotech), Co^2+^ charged immobilised metal affinity chromatography (IMAC), according to the manufacturers guidelines. A batch gravity binding protocol was used followed by a column elution. Briefly, solubolised inclusion bodies were applied to a 500 μL resin bed volume in conical centrifuge tubes and agitated at 4 °C for 30 mins to allow his-tagged proteins to bind. Protein-bound resin was washed 4 times in 8 M urea containing 10 mM imidazole, 300 mM sodium phosphate pH 8.0 and 300 mM NaCl. Resin was then transferred into gravity-flow columns and his-tag proteins eluted in 10 mL 150 mM imidazole and 1 mL fractions collected.

### Protein refolding and analysis

Purified protein fractions were pooled and dialysed against PBS at 32 × volume using a T4 CelluSep tubular cellulose membrane (Fisher Biotech) overnight at 4 °C. A further dialysis round was completed the next day over 4 hours in another 32 × volumes of PBS. Re-folded purified proteins were concentrated to 200 *μ*g/mL using Amicon Filters (Millipore) as per manufacturer’s guidelines

Total amount of purified protein was determined using a bicinchoninic acid (BCA) assay (ThermoFisher Scientific) as per manufacturer’s guidelines. Assays were performed in 96 well plates and 10 μL to 25 μL protein was mixed with 200 μL of working reagent (Reagent A:Reagent B, 50:1). Plates were incubated at 37 °C for 30 min and read on a BMG Labtech Polarstar Omega plate reader at 550 nm. Protein concentration was determined by interpolating values in an equation obtained from the standard curve.

### Mice

Six to eight week C57BL/6 female mice were used for this study. Mice were sourced from the Animal Resources Centre (Perth, WA, Australia). All mice were housed using a Techniplast (Varese, Italy) housing system with unrestricted access to food and water. Mice infected with *M. tuberculosis* were housed in a physical containment level 3 (PC3) facility. Anaesthesia of mice was induced by methoxyflurane inhalation. Euthanasia of mice was done by CO_2_ asphyxiation. The Animal Ethics Committee of the University of Queensland approved all experiments involving mice, in accordance with Australian code for the care and use of animals for scientific purposes.

### Vaccinations

Subunit vaccines and BCG were delivered subcutaneously while mice were anesthetised. Frozen stocks of BCG were diluted to 2.5 × 10^6^ cfu/mL in sterile PBS and 200 μL delivered at the base of the tail (5 × 10^5^ CFU delivered). Doses of BCG delivered was validated by enumerating serial dilutions of the vaccine on 7H11 after three weeks incubation. Recombinant protein vaccines were prepared containing the vaccine antigen (50 *μ*g/mL), dioctadecyl ammonium bromide (DDA) (1.25 mg/mL) and monosphosphoryl lipid A (MPLA) (125 *μ*g/mL) as previously described^19^. DDA was prepared at 2.5 mg/ml in water at 80 °C for 20 to 30 mins with regular agitation. MPLA was prepared at 500 *μ*g/mL in water containing 0.2% triethylamine. MPLA stock was heated to 70 °C for 30 seconds and then sonicated in a water bath sonicator for 30 seconds. This process was repeated 3 times.

Vaccines were emulsified by vortexing for 2 minutes with DDA and MPLA to create the delivered vaccine. 200 μL of the emulsified vaccine was injected subcutaneously at the base of the tail delivering 10 μg of recombinant protein, 500 μg of DDA and 25 μg of MPLA. BCG vaccinated mice were immunised once and recombinant protein vaccinated mice were immunised three times over a four week period before being rested for either four or six weeks.

### ELISpot

Vaccinated mice were sacrificed four weeks post third vaccination and spleens removed. Single cell suspensions were prepared by passing spleens through a 70 *μ*m cell strainer. ELISpot was used to determine the number of antigen specific IFN-γ producing splenocytes as previously described^27^. Antigens were added to wells at final concentrations of Concanavalin A 3 *μ*g/mL, *M. tuberculosis* cell wall protein 10 *μ*g/mL, vaccine antigen 10 *μ*g/mL, 1 *μ*g/mL, and 0.3 *μ*g/mL.

### Protection Assay

Mice were infected with approximately 100 CFU of *M. tuberculosis* H37Rv via the aerosol route using the inhalation exposure system (Glas-Col, Terre Haute, IN) at time points indicated. Cultures were grown to mid-log phase in 7H9 complete media and then washed in PBS + 0.02% tyloxapol. De-clumped suspensions were created by spinning cells at 800 × *g* for 8 min and supernatants were diluted to an optical density at 600 nm of 0.1 to 0.2. 5 mL was nebulised for infection. Post infection mice were rested for four weeks and then sacrificed by CO_2_ asphyxiation. The left lung lobes and spleens were collected and homogenised in 2 mL PBS, serially diluted and plated onto 7H11 agar plates. After 21 days incubation at 37 °C, CFU were counted and bacterial burden on the lungs and spleens determined.

### Histopathology

Bottom right lung lobes were removed from sacrificed mice and fixed in 10% buffered formalin for four weeks. Fixed lobes were paraffin embedded and sections stained with haematoxylin-eosin (HE) and examined by light microscopy on a Zeiss A1 Axio Scope.

### Statement of ethics and accordance

The Animal Ethics Committee of the University of Queensland approved all experiments involving mice, in accordance with Australian code for the care and use of animals for scientific purposes.

## Electronic supplementary material

Supplementary Figures

## References

[1] World Health Organization. Global Tuberculosis Report 2016 (2016).

[2] Fine PEM (1995). Variation in protection by BCG: implications of and for heterologous immunity. Lancet.

[3] Trunz BB, Fine P, Dye C (2006). Effect of BCG vaccination on childhood tuberculous meningitis and miliary tuberculosis worldwide: a meta-analysis and assessment of cost-effectiveness. Lancet.

[4] Arlehamn CSL (2013). Memory T Cells in Latent Mycobacterium tuberculosis Infection Are Directed against Three Antigenic Islands and Largely Contained in a CXCR3+ CCR6+ Th1 Subset. PLoS Pathog.

[5] Lindblad EB, Elhay MJ, Silva R, Appelberg R, Andersen P (1997). Adjuvant modulation of immune responses to tuberculosis subunit vaccines. Infect Immun.

[6] Mbow ML, De Gregorio E, Valiante NM, Rappuoli R (2010). New adjuvant for human vaccines. Curr Opin Immunol.

[7] Holten-Andersen L, Doherty TM, Korsholm KS, Andersen P (2004). Combination of the Cationic Surfactant Dimethyl Dioctadecyl Ammonium Bromide and Synthetic Mycobacterial Cord Factor as an Efficient Adjuvant for Tuberculosis Subunit Vaccines. Infect Immun.

[8] West NP (2011). Delivery of a multivalent scrambled antigen vaccine induces broad spectrum immunity and protection against tuberculosis. Vaccine.

[9] Doherty TM, Weinrich Olsen A, van Pinxteren L, Andersen P (2002). Oral Vaccination with Subunit Vaccines Protects Animals against Aerosol Infection with *Mycobacterium tuberculosis*. Infect Immun.

[10] Kao FF (2012). The Secreted Lipoprotein, MPT83, of *Mycobacterium tuberculosis* Is Recognized during Human Tuberculosis and Stimulates Protective Immunity in Mice. PLoS One.

[11] Korsholm KS, Petersen RV, Agger EM, Andersen P (2010). T-helper 1 and T-helper 2 adjuvants induce distinct differences in the magnitude, quality and kinetics of the early inflammatory response at the site of injection. Immunol.

[12] Garnier T (2003). The complete genome sequence of *Mycobacterium bovis*. Proc. Natl. Acad. Sci. USA.

[13] Domenech P (2004). The Role of MmpL8 in Sulfatide Biogenesis and Virulence of *Mycobacterium tuberculosis*. J Biol Chem.

[14] Dietrich J, Aagaard C, Leah R, Andersen P (2005). Exchanging ESAT6 with TB10.4 in an Ag85B Fusion Molecule-Based Tuberculosis Subunit Vaccine: Efficient Protection and ESAT6-Based Sensitive Monitoring of Vaccine Efficacy. J Immunol.

[15] Aagaard C (2011). A multistage tuberculosis vaccine that confers efficient protection before and after exposure. Nat Med.

[16] Villarreal DO, Walters J, Laddy DJ, Yan J, Weiner DB (2014). Multivalent TB vaccines targeting the esx gene family generate potent and broad cell-mediated immune responses superior to BCG. Hum Vaccines & Immunotherapeutics.

[17] Andersen P, Doherty TM (2005). TB subunit vaccines - putting the pieces together. Microbes and Infection.

[18] Weinreich Olsen A, Hansen PR, Holm A, Andersen P (2000). Efficient protection against *Mycobacterium tuberculosis* by vaccination with a single subdominant epitope from the ESAT-6 antigen. Eur J Immunol.

[19] Shanahan ER, Pinto R, Triccas JA, Britton WJ, West NP (2010). Cutinase-like protein-6 of *Mycobacterium tuberculosis* is recognised in tuberculosisi patients and protects mice against pulmonary infection as a single and fusion protein vaccine. Vaccine.

[20] Weinreich Olsen A, van Pinxteren LAH, Okkels LM, Rasmussen PB, Andersen P (2001). Protection of Mice with a Tuberculosis Subunit Vaccine Based on a Fusion Protein of Antigen 85B and ESAT-6. Infect Immun.

[21] Somvanshi P, Singh V, Seth PK (2008). *In Silico* Prediction of Epitopes in Virulence Proteins of *Mycobacterium tuberculosis* H37Rv for Diagnostic and Subunit Vaccine Design. J Proteomics Bioinfo.

[22] Sievers F (2011). Fast, scalable generation of high-quality protein multiple sequence alignments using Clustal Omega. Molecular Systems Biolocy.

[23] Goujon M (2010). A new bioinformatics analysis tools framework at EMBL-EBI. Nucleic acids research.

[24] Fleischmann, F. D. *et al*. Whole-genome comparison of *Mycobacterium tuberculosis* clinical and laboratory strains. *J Bacteriol* **184**, 5479–5490 (200). 10.1128/JB.184.19.5479-5490.2002 PMC135346 12218036

[25] Krogh A, Larsson B (2001). vonHeijne, G. & Sonnhammer, E. L. L. Predicting transmembrane protein topology with a hidden Markov model: Application to compete genomes. Journal of Molecular Biology.

[26] Weinreich Olsen A, Williams A, Okkels LM, Hatch G, Andersen P (2004). Protective Effect of a Tuberculosis Subunit Vaccine Based on a Fusion of Antigen 85B and ESAT-6 in the Aerosol Guinea Pig Model. Infect Immun.

[27] West NP (2008). Immunological diversity within a family of cutinase-like proteins of *Mycobacterium tuberculosis*. Vaccine.

