# Supplementary material for: Use of Soluble Extracellular Regions of MmpL (SERoM) as Vaccines for Tuberculosis
Source: Sci Rep. 2018 Apr 4;8:5604. doi: 10.1038/s41598-018-23893-3 (PMC5884834; doi:10.1038/s41598-018-23893-3)
Supplement: Supplementary file 1 — Supplementary Figures [file 41598_2018_23893_MOESM1_ESM.pdf]

Use of Soluble Extracellular Regions of MmpL (SERoM) as Vaccines for Tuberculosis

Emily J. Strong [1], Nicholas P. West [1, 2, *]

[1] School of Chemistry and Molecular Biosciences, University of Queensland, Brisbane, 4067, Australia

[2] Australian Infectious Disease Research Centre, University of Queensland, Brisbane, 4067, Australia

[*] Corresponding author: n.west@uq.edu.au

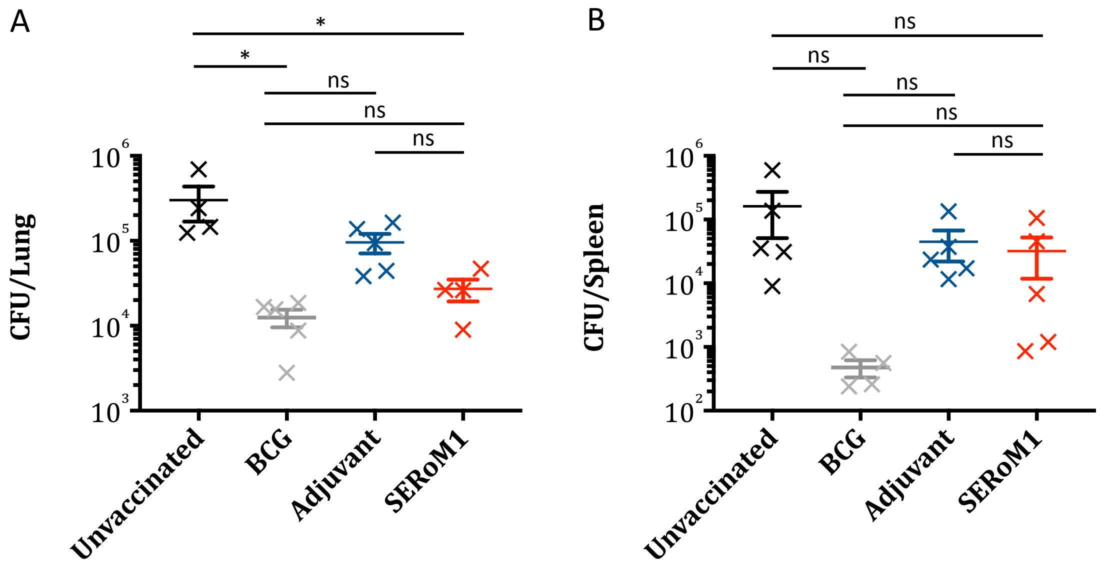

Supplementary Figure 1. Protective effects of SERoM vaccines in C57BL/6 mice. Mice (n=5) were vaccinated subcutaneously three times with SERoM vaccines, adjuvanted with DDA/MPLA. Mice were subsequently aerosol challenged with M. tuberculosis H37Rv 4 weeks post third vaccination. A) protection in the lung of vaccinated mice. B) protection in the spleen of vaccinated mice. Crosses are individual mice with mean and standard error from the mean shown. Significance was calculated compared to relevant control group via one-way ANOVA with Tukey's multiple comparisons test. * $p < 0.05$.

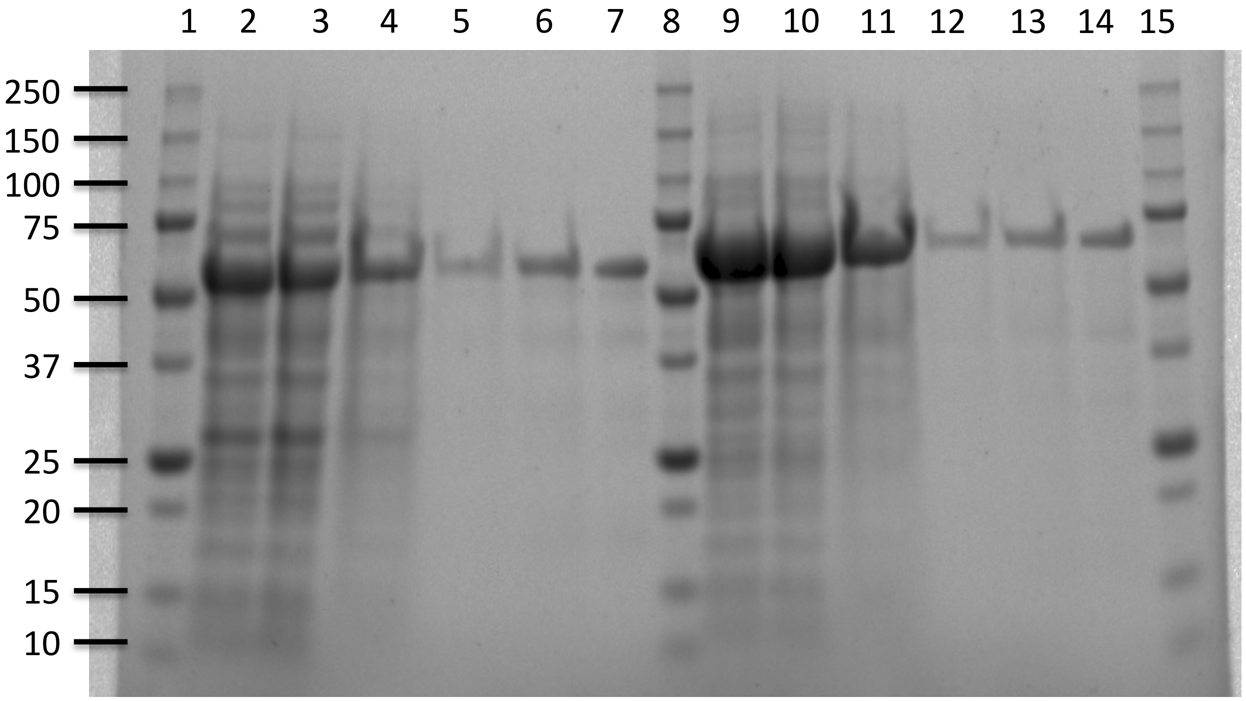

Supplementary Figure 2. SDS-PAGE analysis of purified recombinant vaccine antigens SERoM1 and SERoM8. Lane 1, Molecular Weight Marker; (Lane 2 to Lane 7 SERoM1) Lane 2, Clarified whole cell lysate of *E. coli* SERoM 1 expressing strain; Lane 3, Unbound clarified protein after IMAC purification, Lane 4, 1st IMAC resin wash; Lane 5, 3rd IMAC resin wash; Lane 6, IMAC purified recombinant protein; Lane 7, Purified, refolded soluble MmpL polypeptide. SERoM 1 expected size 58 kDa. Lane 8, Molecular Weight Marker; (Lane 9 to Lane 14 SERoM8) Lane 9, Clarified whole cell lysate of *E. coli* SERoM 8 expressing strain; Lane 10, Unbound clarified protein after IMAC purification, Lane 11, 1st IMAC resin wash; Lane 12, 3rd IMAC resin wash; Lane 13, IMAC purified recombinant protein; Lane 14, Purified, refolded soluble MmpL polypeptide. SERoM 8 expected size 61 kDa. Lane 15, Molecular Weight Marker.

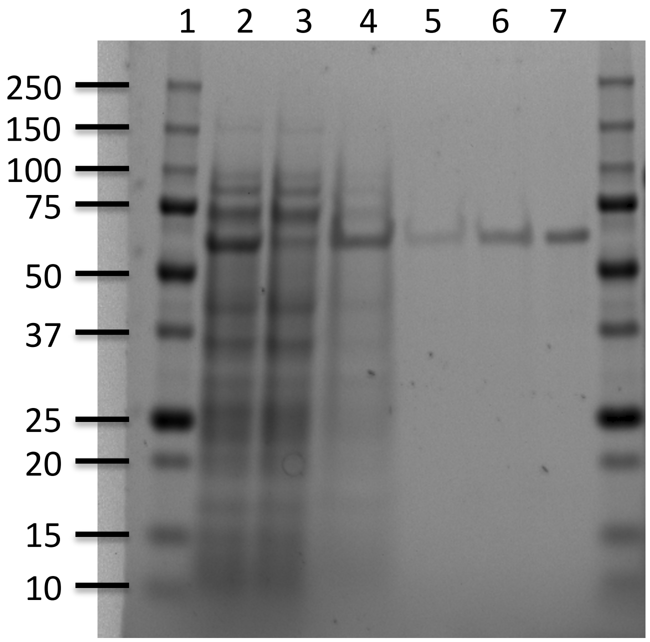

Supplementary Figure 3. SDS-PAGE analysis of purified recombinant vaccine antigen SERoM10. Lane 1, Molecular Weight Marker; Lane 2, Clarified whole cell lysate of *E. coli* expressing strain; Lane 3, Unbound clarified protein after IMAC purification, Lane 4, 1st IMAC resin wash; Lane 5, 3rd IMAC resin wash; Lane 6, IMAC purified recombinant protein; Lane 7, Purified, refolded soluble MmpL polypeptide. Expected size of SERoM10 is 69 kDa.
